# Supplementary figures and images for: Long-term effectiveness and safety of infliximab-biosimilar: A multicenter Phoenix retrospective cohort study
Source: PLoS One. 2023 Sep 12;18(9):e0288393. doi: 10.1371/journal.pone.0288393 (PMC10497130; doi:10.1371/journal.pone.0288393)

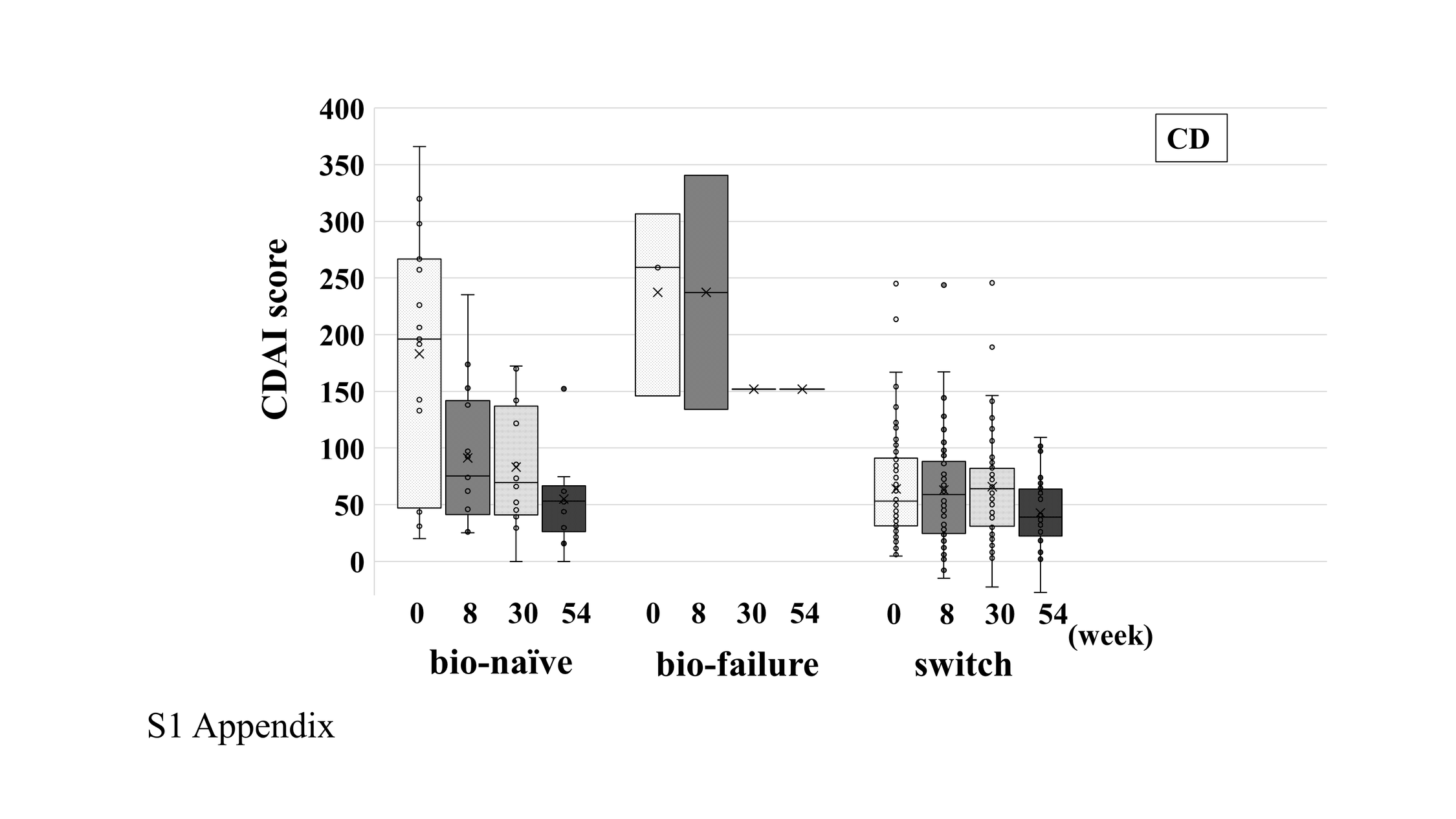

Supplement: S1 Appendix — (TIF) [file pone.0288393.s001.tif]

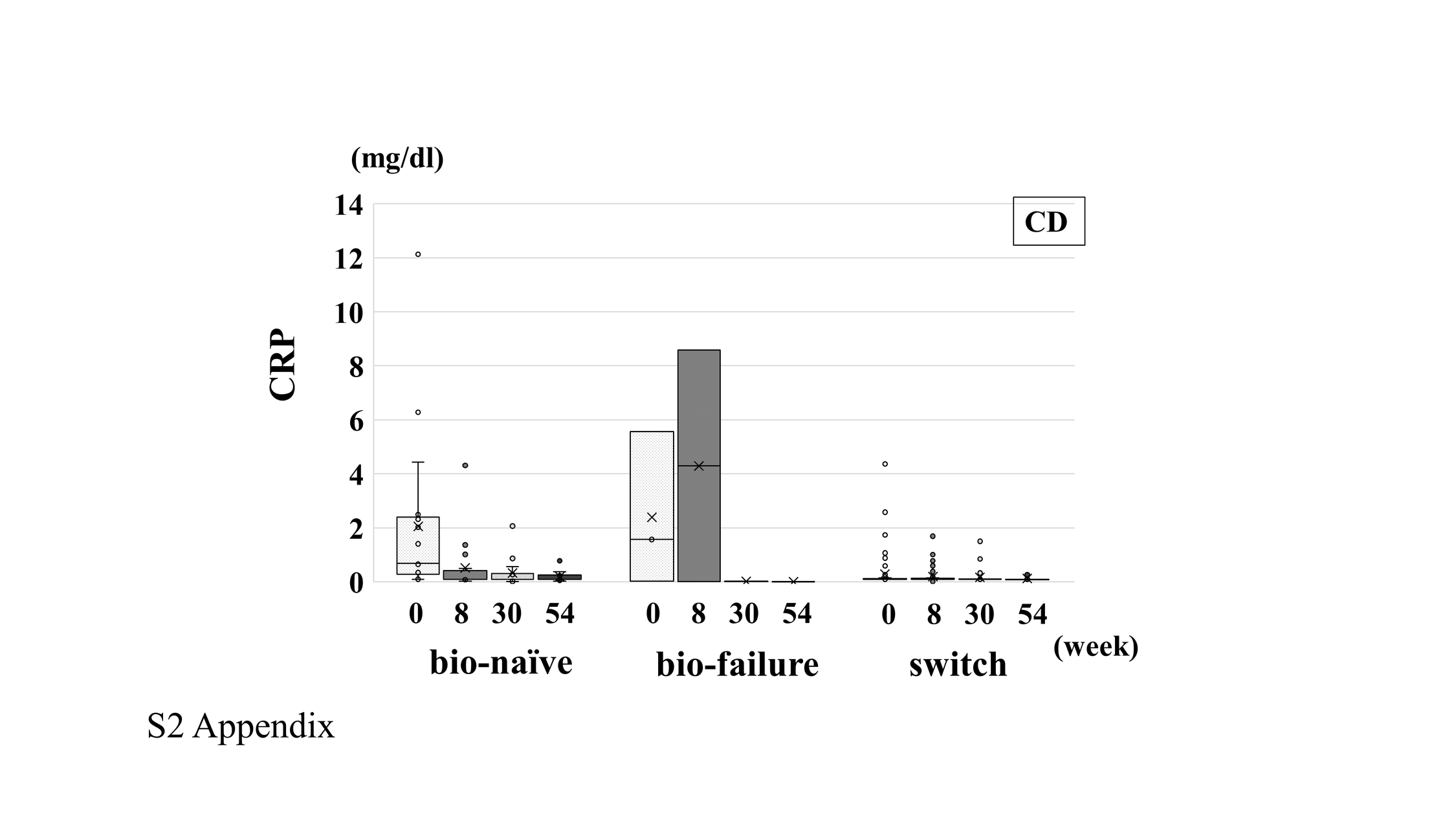

Supplement: S2 Appendix — (TIF) [file pone.0288393.s002.tif]

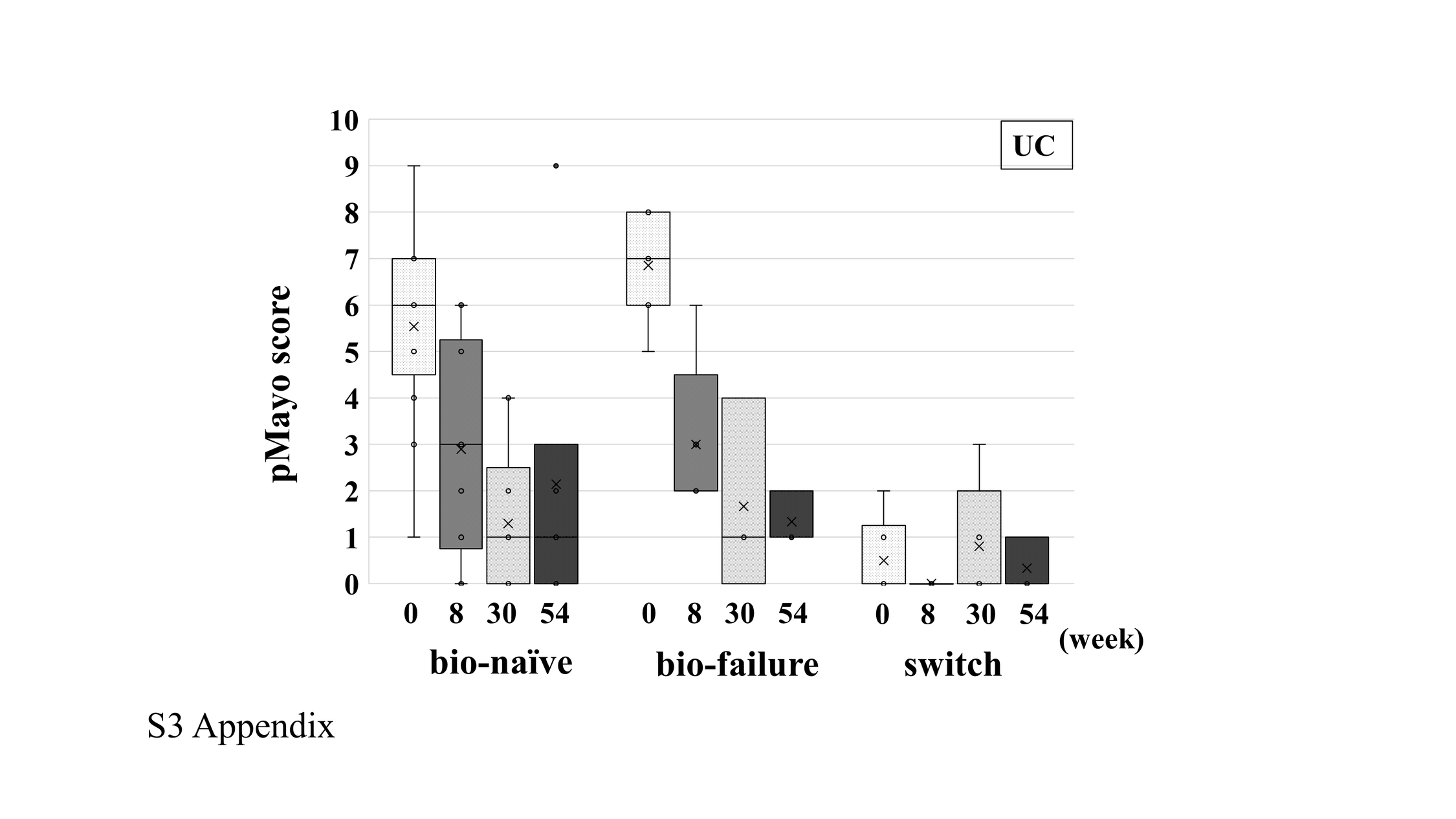

Supplement: S3 Appendix — (TIF) [file pone.0288393.s003.tif]

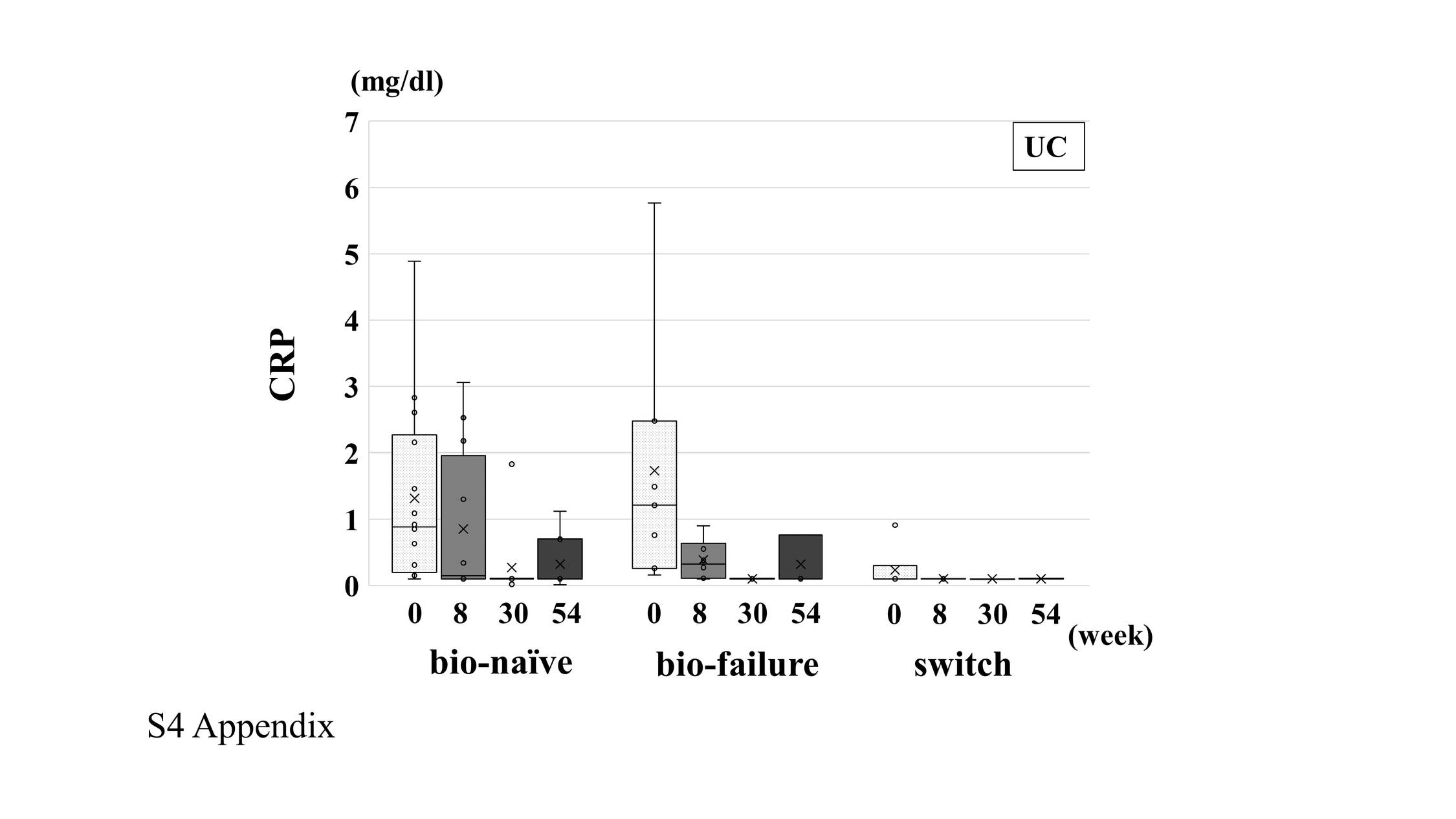

Supplement: S4 Appendix — (TIF) [file pone.0288393.s004.tif]

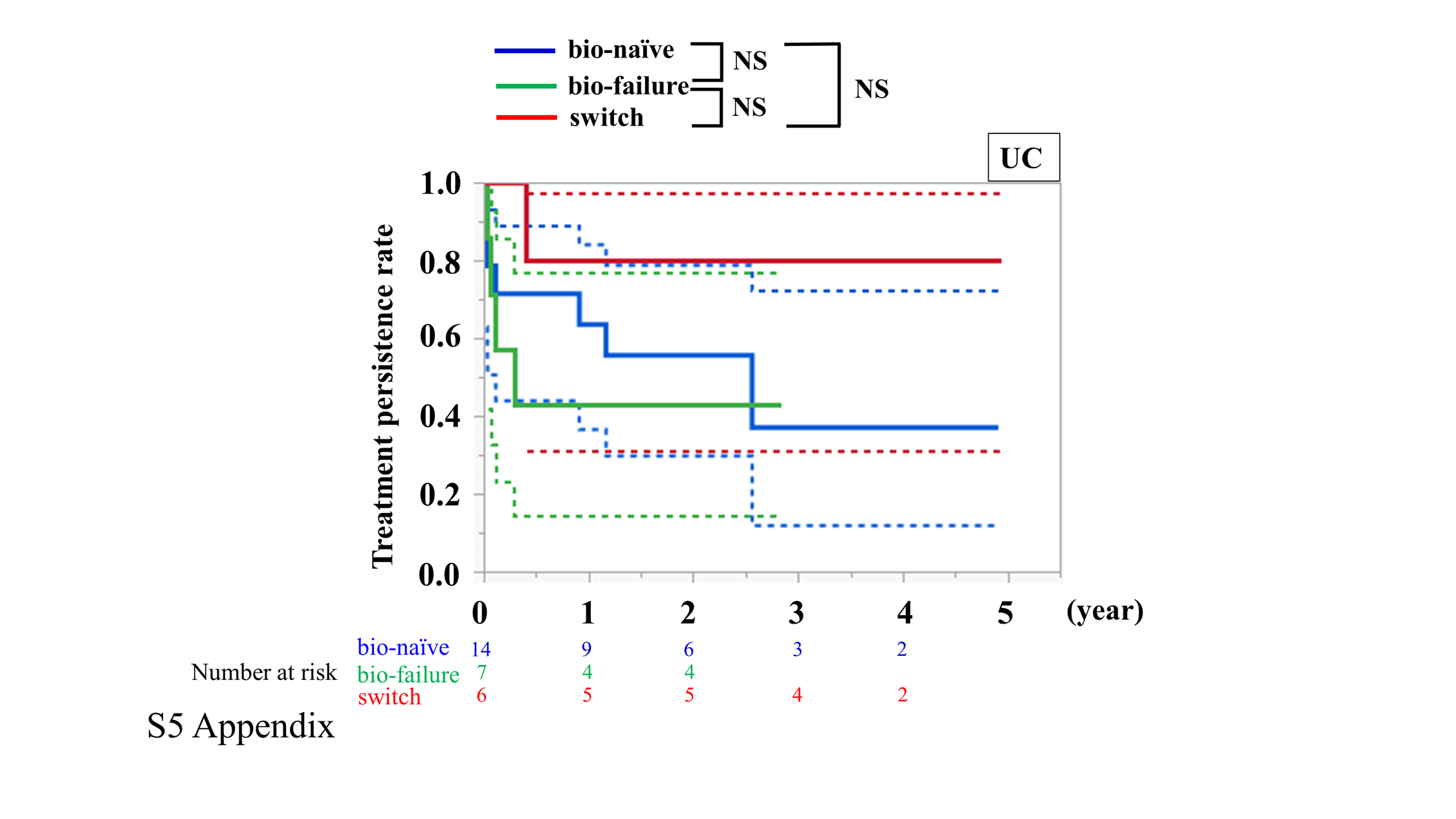

Supplement: S5 Appendix — Statistical significance was analyzed using the log-rank test. NS: Not Significant. (TIF) [file pone.0288393.s005.tif]

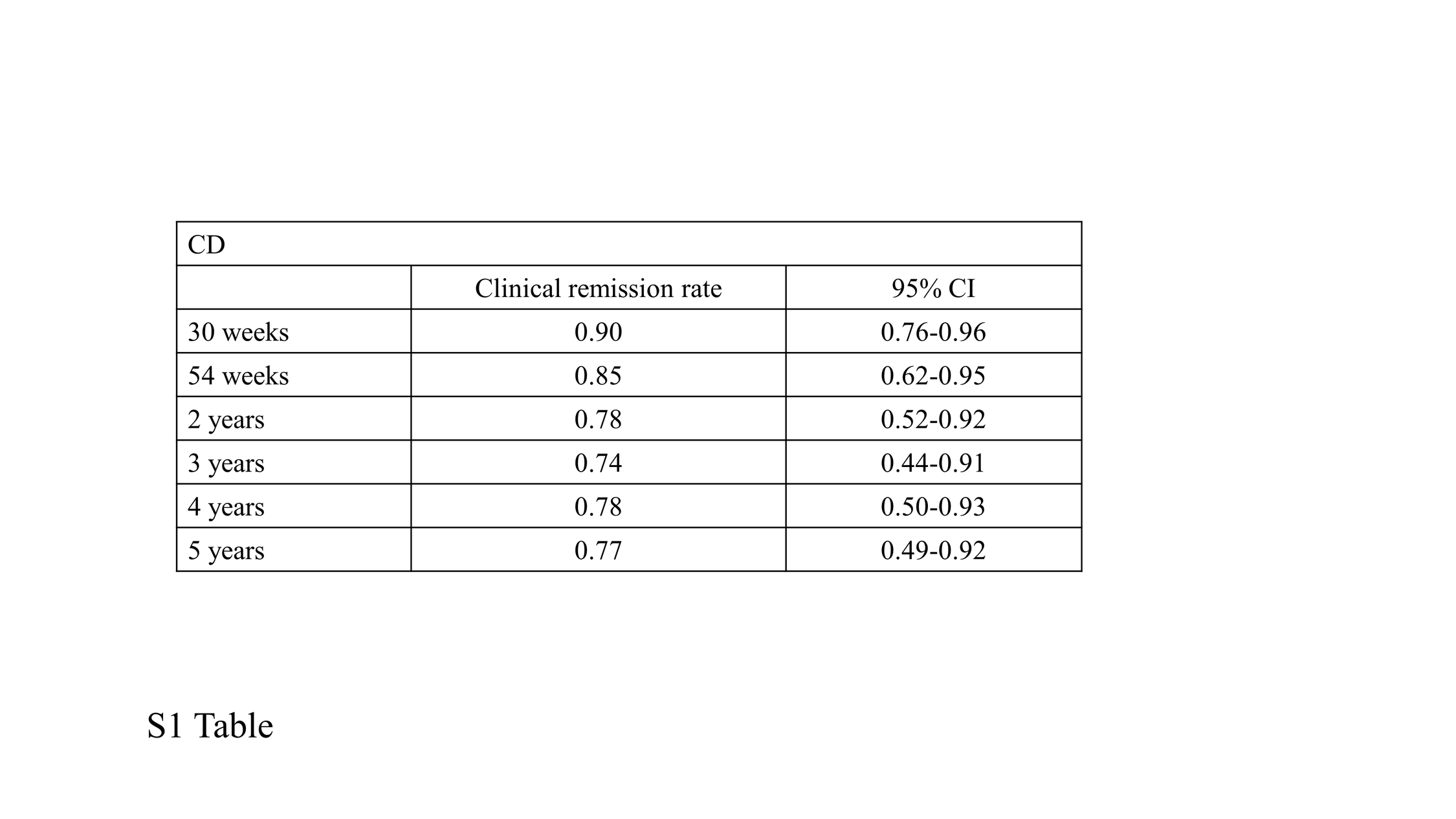

Supplement: S1 Table — (TIF) [file pone.0288393.s006.tif]
