# Supplementary material for: Long-term effectiveness and safety of infliximab-biosimilar: A multicenter Phoenix retrospective cohort study
Source: PLoS One. 2023 Sep 12;18(9):e0288393. doi: 10.1371/journal.pone.0288393 (PMC10497130; doi:10.1371/journal.pone.0288393)
Supplement: Attachment — Submitted filename: Response to the Reviewers.docx [file pone.0288393.s007.docx]

Additional Editor Comments:

-As the Reviewer #1 mentioned, the treatment of missing data is crucial. Please describe in detail in the Methods. Also consider why they are missing, such as whether they have changed treatment or stopped coming to the hospital.

Thank you for your comment. We have added the description about the treatment of missing data in the Materials and Methods section (page 6, lines 126-128):

“Patients who were lost to follow-up or had missed clinical scores were considered censored. In case for which CDAI or pMayo could not be calculated due to missing data, we excluded from subsequent analyses for effectiveness.”

-The persistence rate should vary considerably depending on whether it is Nave, Switch, or Failure. It is insufficient that this variable is not included in the multiple regression analysis. The information of Biologics Failure might be worse predictor than combo therapy.

Thank you for your comment. In this study, there is a limitation of a small number of UC patients. According to the reviewer’ comment, we assessed the factors in favor of the treatment persistence in all CD patients using Cox proportional hazards regression. As the Editor and Reviewer#1 pointed out, each group is expected to have a different potential risk for treatment failure. Therefore, we also performed multivariate analysis restricted to the switch group in CD patients.

-Have you gathered any information on whether the patients used immunomodulator before the baseline or immediately after starting treatment? If you have information when immunomodulator was concomitantly used, please specify. Does the effectiveness change depending on the timing of concomitant use?

Thank you for your comment. We have gathered the information on whether the patients used immunomodulator before the baseline. The concomitant cases are listed in Table 1. However, we did not gathered the information on whether the patients used immunomodulator immediately after starting IFX-BS; therefore, we could not show the change of effectiveness depending on the timing of concomitant use.

-This is likely an unstable model because multivariate analysis was performed despite the small number of no-persistence cases. Since it changes depending on how the missing values were handled, could you also present as supplementary data whether each factor in the Cox proportional hazards was a significant factor? If you do not have the required number of cases, I would be willing to do just a univariate analysis.

According to the Editor’ and Reviewer#1's comment, we used Bayesian methods because we are dealing with a small sample size, and based on the results, we performed a multivariate analysis using Cox proportional hazards regression with concomitant use of immunomodulators and serum albumin as variables.

Reviewer #1:

This study by Kazama et al provides new evidence about the effectiveness of infliximab biosimilars in IBD, in various contexts : at initiation, at medical switch and at non medical switch. Despite the relatively small number of patients included, the authors show different persistence in between groups and highlight predicting factors for persistence under treatment.

Abstract
1. P2 l21 The author mentions « biosimilar » in singular : Japan has not approved more than one biosimilar product for infliximab ? CTP13 ? SB2 ?

Thank you for your comment. In Japan, we can use two biosimilar products for infliximab, Infliximab Biosimilar 1 and Infliximab Biosimilar 3. We have correct to plural where necessary and revised Materials and Methods section as follow (page 4, line 79-83):

“We enrolled CD and UC patients who received IFX-BS (Infliximab Biosimilar 1,commercialized at Nov 2014, Nippon Kayaku Co., Ltd. Tokyo, Japan, and Infliximab Biosimilar 3, commercialized at Dec 2018, Pfizer Inc., NY, USA) from October 2016 to April 2022.”

2. P2 It would be good to add the dates of commercialization of infliximab originator and biosimilar products.

Thank you for your suggestion. We have added the dates of commercialization of infliximab originator and biosimilar products in the Materials and Methods section as follows (page 3, lines 55-58, and page 4, line 79-83):

“Anti-TNF-α agents, such as infliximab (IFX, commercialized at May 2002, Mitsubishi Tanabe Pharma Corporation, Osaka, Japan), are highly effective in the induction and maintenance of remission in IBD and have remarkably improved patients’ quality of life (1, 2).”

“We enrolled CD and UC patients who received IFX-BS (Infliximab Biosimilar 1, commercialized at Nov 2014, Nippon Kayaku Co., Ltd. Tokyo, Japan, and Infliximab Biosimilar 3, commercialized at Dec 2018, Pfizer Inc., NY, USA) from October 2016 to April 2022.”

3. P2 l38 Typo : findngs => findings

Thank you for your kind suggestion. We have corrected the typo (page 2, line 38).

4. P2 It would have been interesting to compare the effectiveness and adverse events rates in patients taking the originator product. The authors could at least cite the persistence rates known for the originator product.

Thank you for your comment. Unfortunately, it is difficult to compare the effectiveness and adverse event rates in patients taking the originator products because we have no data available. Instead, we have added the persistence rates of the infliximab originator, which has been reported in previous reports, in the Discussion section (page 19, lines 287-291):

“Blesl et al. reported that the 5-year persistence rate of IFX originator for CD was 39% (95% CI: 34-45), and Masui et al. reported it was 62.9% (95% CI: 55.8–69.4) (26, 27). Despite a wide range of the persistence rate reported previously, the persistence rate of IFX-BS in this study was not inferior to that of the originator.”

Introduction
1. P4 l62 : IFX-BSs are not approved for psoriasis ?

Thank you for your comment. In Japan, IFX-BSs are approved for psoriasis. We have revised the sentence as follows (page 3, line 60 - page 4, line 62):

“They are approved for use in rheumatoid arthritis (RA), ankylosing spondylitis, CD, UC, and psoriasis.”

2. Please comment on the regulatory process for biosimilar approval and the specific process in Japan.

In Japan, biosimilars are approved by extrapolation. In case that the original product has multiple indications and that clinical studies confirm the equivalence to the original product in a certain disease, other indications can be approved without clinical studies. Regarding infliximab, biosimilars were approved for UC and CD based on the result of clinical studies for rheumatoid arthritis.

Material and Methods
1. P4 l81 : as the objective is to assess the long term persistence of patients, why didn’t the authors stopped the inclusions at least 1 or 2 years before the end of study ?

Thank you for your comment. As the reviewer pointed out, this retrospective cohort includes cases in which the observation period is relatively short. We have added the observation period in Table1. The efficacy and safety of IFX-BS should be further clarified in prospective studies with a sufficient observation period after inclusion.

2. P5 l90 : what is the difference between the biofailure group that took IFX originator or adalimumab and the switch group ? Could the switch other anti-TNF alpha to IFX BS be non medical and comparable to the switch originator IFX to IFX BS ?

Thank you for your comment. We defined the bio-failure group as patients with primary or secondary ineffectiveness or adverse events to the originator that enabled them to continue treatment. The switch group is defined as patients with stable disease but switched to biosimilars.

In this study, we did not include the patient who switched from other anti-TNF-α inhibitors to biosimilars for non-medical reasons.

3. P5 L96 : How did you deal with lost of follow-up ? with censoring ?

Thank you for your important comment. In this study, patients who were lost to follow-up or had missed clinical scores were considered censored. We have added the following sentence in Materials and Methods section (page 6, lines 126-127):

”Patients who were lost to follow-up or had missed clinical scores were considered　censored.”

4. P6 l111 : define the CR here (it is written on line 113)

Thank you for your suggestion. We have revised the sentences in the Materials and Methods section as follows (page 6, lines 114-118):

“In patients with CD, clinical remission (CR) was defined as Crohn’s disease activity index (CDAI) <150 points, and clinical response as a decrease of at least 70 CDAI points from baseline. In patients with UC, CR was defined as a partial Mayo (pMayo) score of two points or fewer, and clinical response was defined as a decrease in the pMayo score by at least 30% from baseline.”

5. P6 L117 : “We also evaluated the clinical response in the bio-naïve and bio-failure groups” this sentence is unclear : clinical response is not evaluated in all the groups ? the beginning of the paragraph seemed to say so…

Thank you for your comment. In this study, we evaluated the clinical response in the bio-naïve and bio-failure groups. On the other hand, we evaluated the clinical remission in switch group. We administered biosimilars to patients in remission for non-medical reasons; therefore, it is considered appropriate to evaluate the maintenance of remission rather than the response. I apologize to confuse you. We have revised the sentence in the Materials and Methods section as follows (page 6, lines 118-119):

“Endpoints of this study were the proportion of patients achieving CR in the switch group and persistence of long-term administration of IFX-BS.”

6. P6 L117 : can you please define the events of interest taken into account for safety ?

Thank you for your comment. For the safety profile of IFX-BS, all patients were asked about adverse events, including infusion reactions, infection, hospitalization, and any potential adverse events at every visits. We have added the following sentence in the Materials and Methods section (page 6, lines 124-126):

“For the safety profile of IFX-BS, all patients were asked about adverse events, including infusion reactions, infection, hospitalization, and any potential adverse events at every visits.”

7. P6 L120 : Please define CRP

We have described the full spelling of CRP where it first appeared (page 13, line 172).

8. P6 L128 : as the authors are modelling persistence time, wouldn’t it have been appropriate to model the risk of non persistence with a Cox regression or a Poisson regression ? as the authors are dealing with small sample sizes, wouldn’t it have been appropriate to use Bayesian methods ?

Thank you for critical comments. We have assessed the factors in favor of the treatment persistence using a Cox regression. We also used Bayesian methods. The results of Cox regression analysis is shown in Table 2, and Bayesian analysis is shown in Fig 4.

9. Please cite the “Kaplan Meier” methodology for persistence modelling.

Thank you for your comment. We have added the following sentence in the Materials and Methods section (page 7, lines 137-138):

“Treatment persistence was assessed by the Kaplan-Meier method and Wilcoxon test using JMP^®^ Pro (version 16.2.0, SAS Institute, Cary, NC).”

10. P7 l129 : please clarify the methodology for the logistic regression modelling the factors in favor of persistence : do you compare “at least 1 year persistent patients” to “less than 1 year persistent patients” ? Did you carry out sensitivity analyses on 2/3/4/5 years persistent patients ? Did you try to look at low / medium / high persistent patients ?

Thank you for your comment. According to your valuable comment, we have assessed the factors in favor of treatment persistence using Cox proportional hazards regression instead of logistic regression. We have added the sentence in the Materials and Methods section as follows (page 7, lines 133-136):

“Cox proportional hazards regression were performed with EZR v1.61 (Saitama Medical Center, Jichi Medical University, Saitama, Japan), which is a graphical user interface for R (The R Foundation for Statistical Computing, Vienna, Austria)”

Results
1. Table 1 : What does “range” mean ? interquartile range ? min-max range ?

Thank you for your comment. We showed max-min range in the first draft, but we have revised the table using inter quartile range (IQR).

2. Table 1 : back to the methods “The switch group included patients who received IFX originator or other anti-TNF-α agents in remission at baseline and switched to IFX-BS” : finally, the switch group is exclusively composed of IFX originator => IFX BS switchers right ?

Thank you for your comment. As the reviewer mentioned, the switch group is composed of patients in remission using IFX-originator, not contains the patients switched from other TNF-α agents. We have revised the sentence in the Materials and Methods section as follows (page 5, lines 92-93):

“The switch group included patients who received IFX originator in remission at baseline and switched to IFX-BS.”

3. Table 1 (and other results) : as your sample size is quite small, please consider using only 2 significant digits in the percentages calculations.

Thank you for your comment. We have revised Table1 using only 2 significant digits in the percentages calculations.

4. Table 1 : please add the year of inclusion

Thank you for your comment. We have added the year of inclusion in Table 1.

5. Table 1 is not organized like state of the art Table 1, and thus quite hard to read.

Thank you for your comment. We have revised Table 1 to comfortable format.

6. Figures : CDAI score, CRP, and pMayo score can be put in supplementary material

Thank you for your comment. We have transferred Figures 2A-B and 4A-B to supporting information S1-4, and other figure numbers have been changed accordingly.

7. Figure legends : please specify in the legend the group of patients which is considered. CR was evaluated for switch group only ? Please clarify in the Methods section.

Thank you for your comment. We have added the number of patients at risk in the bottom part of the new Fig 3 and S5 Appendix. In this study, clinical remission (CR) was evaluated for switch group. We have added the sentence in the Materials and Methods section as follows (page 6, lines 118-119):

“Endpoints of this study were the proportion of patients achieving CR in the switch group and persistence of long-term administration of IFX-BS.”

8. Is it relevant to show the results of CD and UC separately ? The number of UC patients is very small and thus at risk of sampling fluctuation.

Thank you for your comment. As the reviewer pointed out, there is a risk of sampling fluctuation due to the small number of UC cases. However, the pathologies, treatment options, and treatment efficacy with IFX differ between UC and CD. Therefore, we decided to analyze UC and CD separately.

9. Kaplan meier curves : please show the number of patients still at risk throughout the follow-up period. Please add the confidence intervals / bands. This seems to be important figures to highlight more.

Thank you for your comment. We have added the number at risk in the bottom part of the new Fig 3 and S5 Appendix, and showed the confidence intervals and bands.

10. P12 l199 : please show the confidence intervals for persistence rates

Thank you for your comment. We have added the confidence intervals for persistence rates in Result section and Figure legends

11. Table 2/3 : please consider using a model with UC and CD patients together, adjusting on the pathology

Thank you for your comment. In the present study, we did not check the pathological findings when inducing or switching to IFX-BS. Therefore, unfortunately, we could not analyze using the model reviewer suggested.

12. Table 2/3 : how did you categorized the variables hemoglobin, albumin, CRP ? Is it based on clinical thresholds ?

Thank you for your question. In the first draft, we have divided the patients into two groups using the median value of hemoglobin, albumin, and CRP. However, in the revised version, we performed Bayesian model averaging and Cox proportional hazards regression analysis. Alb was used as continuous variable.

13. Table 2/3 : please show the effectives in each category and the shares of persistent vs non persistent

Thank you for your suggestion. As the assessment of treatment effectiveness, we showed responder rate in bio-naïve and bio-failure group in Fig 1A and new Fig 2A. We also showed clinical remission rate in switch group in Fig 1B and new Fig 2B. To show the share of persistent and non-persistent groups in each category, a number at risk was added to the Kaplan-Meier curve (Fig 3 and S5 Appendix).

14. Please show the number of person years, mean and median follow up durations, for each group and each disease

Thank you for your comment. We have added the number of person years, mean and median follow up durations for each groups in Table 1.

15. P13 L220 : no conclusion can be drawn on UC patients

Thank you for your comment. As the reviewer indicated, due to the small number of UC patients, we could not draw any conclusions about UC patients. In the revised manuscript, we assessed the factors in favor of treatment persistence only in CD patients.

16. P16 l240 : please show the incidence of adverse events relatively to the follow up duration

Thank you for your comment. As the reviewer pointed out, the onset of adverse events is clinically important issue. However, unfortunately, we did not collect information on the date of onset of adverse events in this cohort.

Discussion
1. P17 l252 : as the authors did not compare head to head biosimilar and originator, the statement “We found that the effectiveness of remission induction and maintenance by IFX BS was comparable to that by IFX originator” is misleading and exaggerated

Thank you for your comment. As the reviewer pointed out, we did not compare head-to-head biosimilar and originator. We have revised the sentence in the Discussion section as follows (page 18, lines 255-256):

“We found that the effectiveness of remission induction and maintenance by IFX-BS was comparable to that by IFX originator.”

2. P17 L255 : the CRP was not strictly significantly associated with persistence

Thank you for your comment. We have assessed the factors in favor of treatment persistence using Cox proportional hazards regression analysis. As a result, higher albumin levels and concomitant use of immunomodulators were significantly associated with treatment persistence in CD patients. We have added the following sentences in the Results section (page 15, lines 223-228).

“Multivariate analysis for persistence rates in all patients with CD showed that high albumin levels (HR=0.36, 95% CI: 0.15-0.86, P=0.021) were significantly associated with treatment persistence. Although there is no statistical significance, concomitant use of immunomodulators tended to correlate with treatment persistence in all patients (HR=0.43, 95% CI: 0.17-1.09, P=0.076) and switch group (HR=0.31, 95% CI: 0.085–1.10, P=0.070) in CD patients.”

3. P17 l261 : the figure for France in incorrect
<https://www.nature.com/articles/s41598-022-24050-7> ; <https://www.sciencedirect.com/science/article/abs/pii/S0040595721002596?via%3Dihub>

Thank you for your suggestion. We have shown the latest figure of market share in France and South Korea, and added the relevant citations.

4. P18 l 275 : “One possible reason for the bio-failure group in UC also showing a better response to IFX-BS as compared to that in CD is the difference in the prior treatment. IFX-BS can be effective in patients who switched from adalimumab because of the increase in antibody agent according to patient weight, resulting in a relative increase in treatment intensity. In addition, IFX-BS can be effective in cases of LOR with an anti adalimumab antibody. Furthermore, in cases previously treated with vedolizumab or tofacitinib, IFX-BS was the first anti-TNF-α agent and can be effective” is there some literature on this topic ?

Thank you for your comment. Although several case reports showed the effectiveness of IFX originator or IFX-BS for patients previously treated with vedolizumab or tofacitinib, we could not find any literature on the specific mechanism. We have added the literature about the effectiveness of IFX in CD patients after adalimumab failure.

5. P18 l282: I am not very comfortable with your statement, as bio naïve patients are by construction more subject to treatment failure than switching patients that are stabilized by the originator treatment… Once again, you did not compare switchers with non switchers, so you cannot conclude unfortunately.

Thank you for your comment. As you point out, we did not compare switchers and non-switchers. We have deleted the relevant sentence in the Discussion section.

6. P19 l305 : “There can have been a bias in the cases” please clarify

Thank you for your comment. In this study, the sample size was relatively small, especially in UC patients and the bio-failure group. We have changed the sentences in the Discussion section as follows (page 20, lines 310-313):

“First, the sample size was relatively small, especially in UC patients and the bio-failure groups with CD and UC patients. UC patients has more treatment options, which could be why we had a few UC patients in this retrospective cohort data.”

7. P19 l305 “switch group was expected to have included more patients with CD at the time of IFX administration” please clarify

Thank you for your comment. In general, UC patients have more treatment options than CD patients, which could be why we had a few UC patients in this retrospective cohort data. We have revised the sentence in the Discussion section as follows (page 20, lines 310-313):

“First, the sample size was relatively small, especially in UC patients and the bio-failure groups with CD and UC patients. UC patients has more treatment options, which could be why we had a few UC patients in this retrospective cohort data.”

8. Limitation : study restricted to 3 japanese centers

Thank you for your comment. We have added the following sentences in the Discussion section (page 20, lines 313-315):

“Second, our data are from only three facilities in Japan. A multicenter study with a larger sample size is needed to evaluate the long-term efficacy and safety of IFX-BS.”

Reviewer #2:

It is my pleasure to review the manuscript entitled "Long-term effectiveness and safety of infliximab-biosimilar: a multicenter Phoenix retrospective cohort study".
However, I have several concerns that need to be addressed before considering publication.

1) The authors write that "Univariate analysis for persistence rate demonstrated that concomitant use of immunomodulator (OR=3.90, 95% CI: 0.77-19.70, P=0.09) and low CRP levels during initial administration of IFX-BS (OR=3.97, 95% CI: 1.04-15.14, P=0.04) were associated with persistence in patients with CD." on line 214-217, page 13. I think "concomitant use of immunomodulator" is not significantly associated with persistence in patients with CD.

Thank you for your comment. We assessed the factors in favor of treatment persistence using Cox proportional hazards regression and found that serum albumin levels significantly associated with persistence in patients with CD. We have revised the sentence in the Results section as follows (page 15, lines 223-225):

“Multivariate analysis for persistence rates in all patients with CD showed that high albumin levels (HR=0.36, 95% CI: 0.15-0.86, P=0.021) was significantly associated with treatment persistence.”

2) The authors show the responder rate in patients with UC at baseline, 8, 30, and 54 weeks in Figure 3A. In bio-naive group, the denominator at 8 weeks is 11. And the denominator at 30 weeks is 14. It is unnatural that denominator increases over time. Please check the study data.

Thank you for your comment. We excluded several cases for which CDAI or pMayo could not be calculated due to missing data from subsequent analyses for effectiveness. We have added a description of how to deal with missing data in the Materials and Methods section as follows: (page 6, lines 127-128)

“In cases for which CDAI or pMayo could not be calculated due to missing data, we excluded them from subsequent analyses for effectiveness.”

We have also revised the patient numbers in the new Fig 1B and Fig 2A.

3) I think it might be better to mention the duration of administration of IFX-BS and observation period of each group (All CD patients, bio-naïve group with CD, bio-failure group with CD, switch group with CD, All UC patients, bio-naïve group with UC, bio-failure group with UC, switch group with UC).

Thank you for your comment. We have added the observation period of each group in Table 1.
