# Supplementary material for: Long-term effectiveness and safety of infliximab-biosimilar: A multicenter Phoenix retrospective cohort study
Source: PLoS One. 2023 Sep 12;18(9):e0288393. doi: 10.1371/journal.pone.0288393 (PMC10497130; doi:10.1371/journal.pone.0288393)
Supplement: Attachment — Submitted filename: Response to reviewers.docx [file pone.0288393.s008.docx]

**Additional Editor Comments:**
*[While the authors assume that missing clinical scores are censored, it is generally more appropriate to perform the multiple-imputation method. Furthermore, it's essential to acknowledge the potential bias that may arise from treating a missing score as censored.]*

Thank you for your important suggestion. Due to the frequent missing of follow-up, we additionally applied the inverse probability of censoring weighting (IPCW) method for clinical remission rates in the switch group of CD. The results are shown in S1 Table , demonstrating a higher remission rate than Fig 1B. In this cohort, almost all patients of censored cases were continuable for IFX-BS, which could be the reason of the difference between the original data and IPCW method. Therefore, as the editor pointed out, the treatment of missing scores as censored could be the potential bias.

We have added the following sentences in the Materials and Methods section (page 7, lines 134-144), the Results section (page 14, lines 214-217), and the Discussion section (page 21, lines 346-351):

“Due to the frequent missing of follow-up, we additionally applied the inverse probability of censoring weighting (IPCW) method to estimate the probability of the clinical remission rate in CD patients for each time point. The probability of censoring was estimated using baseline measurements (sex, clinical disease type, prior medications, dose escalation of prior anti-TNF-α inhibitor medication, shortened duration of prior anti-TNF-α inhibitor therapy, nutritional therapy, concomitant use of 5-ASA, steroids, and immunomodulators, albumin level, hemoglobin level, CRP level, CDAI score, history of intestinal resection, IFX-BS dose escalation, shortened IFX-BS duration) using a logistic regression model. We then estimated the remission rate by weighting the inverse probability of uncensored until each time point using generalized estimation equation with robust variance estimates.”

“The results of the clinical remission rates in the switch group with CD patients corrected by the IPCW method are shown in S1 Table. CR rates at 30 weeks, 54 weeks, 2 years, 3 years, 4 years, and 5 years were 0.90 (95%CI: 0.76–0.96), 0.85 (0.62–0.95), 0.78 (0.52–0.92), 0.74 (0.44–0.91), 0.78 (0.50–0.93), and 0.77 (0.49–0.92), respectively (S1 Table). Clinical remission rates were improved in comparison to those before the IPCW analysis.”

“The clinical remission rates for the switch group of CD were also analyzed using the IPCW method (S1 Table). The results showed a higher remission rate than that of Fig 1B. In this cohort, almost all patients of censored cases were able to continue IFX-BS treatment, which could be the reason for the difference between the original data and data obtained by the IPCW method. The treatment of missing scores as censored could potentially lead to bias.”

*[The Kaplan-Meier method usually uses the log-rant test instead of the Wilcoxon test because the log-rank test is generally more powerful and appropriate for detecting differences between groups with respect to time-to-event outcomes. Could you explain why you choose the Wilcoxon test?]*

Thank you for your comment.

We considered that the longer duration of IFX-BS administration, the fewer patients would continue IFX-BS. Also, the number of cases was relatively small in this cohort. Therefore, we considered a generalized Wilcoxon test appropriate and could focus on data from the first half of the study periods.

However, since the log-rank test is commonly used in survival analysis, we used the log-rank test in the revised manuscript. The Kaplan-Meier analysis for persistent rates in CD and UC patients was shown in Fig 3 and S5 Appendix, respectively.

**Reviewer #1:**

*[1) Change “multivariate” for “multivariable”]*

Thank you for your comment. We have changed “multivariate” to “multivariable”. (page 3, line42; page7,line148; page 19, line292)

*[2) Please state the variables (and units / categories) included in the multivariable cox regressions models at the bottom of table 2]*

Thank you for your suggestion. These variables (and units / categories) are listed in new Table 4-5.

*[3) Change “predictors” for “covariates associated with”]*

Thank you for pointing that out.　We have changed “predictors” to “covariates associated with”.（new Table 4-5）

*[4) For the Bayesian method, I was simply suggesting to use Bayesian methodology to reduce : introducing a prior distribution for the HR / OR, calculating the posterior distribution, getting credibility intervals rather than 95% CI that are not relevant for small populations… I find the BMA quite confusing, maybe not explained enough.]*

Thank you for your comments, and we apologize for the confusion caused by our incorrect understanding. In order to examine the Bayesian method, data from similar studies are required for a prior distribution. However, in this study, we could not prepare previously published data examining 5-year persistence rates. Therefore, we could not perform the analysis using the Bayesian method. We have deleted the Figure and descriptions about BMA. Instead, we performed Cox regression analysis on covariates associated with persistence rates in CD patients. The results are shown in new Table 4-5.

We have added the following sentences in the Result section (page 15-16, lines 249-256) and the Discussion section (page 20-21, lines 333-337):

“Univariable analysis for treatment persistence rates in all patients with CD showed that albumin level (HR=0.36, 95% CI: 0.15–0.90) was significantly associated with treatment persistence.

Although multivariable analysis adjusted for age and sex for treatment persistence rates in all patients with CD showed that albumin level (HR=0.40, 95% CI: 0.16–1.01) was not significantly associated with treatment persistence, it was well consistent with the result of the univariable analysis. No other significant covariates contributing to the treatment persistence rates were identified (Table 4 and 5).”

“Univariable analysis of treatment persistence rate showed that albumin level at the initial administration of IFX-BS was significantly associated with treatment persistence. No significant difference in albumin level was observed according to multivariable analysis adjusted for age and sex, but as the HR did not change a lot, it is thought to have contributed to the treatment persistence rate.”

*[5) Figures are displayed in low quality ?]*

Thank you for your comment. We have checked the quality of Figures and improved the resolution.

*[6) Table 1 : split into 2/3 tables, not to mix the group data (naïve/failure/switch) and the complete data]*

Thank you for your comment.　We have divided the Table 1 into three tables (Table 1-3), and have revised the sentence in the Results section as follows (page 8, line 159-160):

“A total of 117 patients with IBD (90 CD and 27 UC) were included. The baseline characteristics of the included patients are shown in Table 1, 2, and 3.”

*[7) When displaying 95% CI, do not show p values]*

Thank you for your comment. P values have been removed from new Table 4-5.

*[8) In patients with CD, persistence rates at 2, 3, and 5 years were 68.8% (95%CI: 31.6-91.3), 68.8% (31.6-91.3), 68.8% (4.8 years, 31.6-91.3) in the bio-naïve group, and 77.6% (66.1-86.1), 66.0% (51.5-78.1), 66.0% (51.5-78.1) => weird to show 3 times the same persistence rates => show only the 2-year persistence (not enough patients to state the 5 years persistence)]*

Thank you for your comment. We have revised the sentence in the Results section as follows (page 15, line 233-234):

“In patients with CD, treatment persistence rates at two years were 85.9% (95%CI: 57.4–96.5) in the bio-naïve group, and 86.0% (75.0–92.6) in the switch group, respectively.”

*[9) “In patients with UC, the persistence rates at 2, 3, and 5 years were 43.1% (95%CI: 19.0-71.0), 43.1% (11.4-71.0), 43.1% (11.4-71.0) in the bio-naïve group, and 66.7% (26.8-91.6), 66.7% (26.8-91.6), and 66.7% (26.8-91.6) in the switch group,” => weird to show 3 times the same persistence rates => show only the 2-year persistence (not enough patients to state the 5 years persistence)]*

Thank you for your comment. We have revised the sentence in the Results section as follows (page 15, line 238-240):

“In patients with UC, the treatment persistence rates at two years were 55.6% (95%CI: 29.7–78.7) in the bio-naïve group, and 80.0% (30.9–97.3) in the switch group, indicating numerically higher treatment persistence rates in the latter group (S5 Appendix).”

**Reviewer #2:**

*[1 Some graphs indicate the result of CD patients and the others indicate the result of UC patients. I found it a little confusing. How about add simple explanations to the figures?]*

Thank you for your comment. We have added the description “CD” or “UC” for each figure. (Fig 1A-3, Table 4-5, S1 Appendix-S5 Appendix, S1 Table)

*[2 Boxplots in appendix are difficult to understand. What do the white circles mean?]*

Thank you for your comment. The white circles mean the data measured at each time point.

*[3 Typo : On the contary => On the contrary (page 8, lines 168)]*

Thank you for your comment. We have corrected the typo (page 8, line 169-170).

*[4 Typo : showen => shown (page 14, lines 213)]*

Thank you for your comment. We have corrected the typo (page 15, line 244).

*[5 Typo : Kissei Pharmaceutidal Co., Ltd. => Kissei Pharmaceutical Co., Ltd. (Competing Interests)]*

Thank you for your comment. We have corrected the typo.
